# Optimal Selenium Fertilizer Affects the Formation of Foxtail Millet ( *Setaria italica* L.) Quality by Regulating Flavonoid Metabolism and Amino Acid Metabolism

**DOI:** 10.1002/fsn3.70362

**Published:** 2025-05-30

**Authors:** Shu Wang, Jiao Mao, Yuanmeng Xu, Sichen Liu, Zhijun Qiao, Xiaoning Cao

**Affiliations:** ^1^ Center for Agricultural Genetic Resources Research Shanxi Agricultural University Taiyuan China; ^2^ College of Agriculture Shanxi Agricultural University Jinzhong China; ^3^ Key Laboratory of Crop Gene Resources and Germplasm Enhancement in the Loess Plateau Ministry of Agriculture and Rural Affairs Taiyuan China

**Keywords:** foxtail millet, metabolomics, quality, selenium

## Abstract

In order to clarify the regulation mechanism of selenium (Se) on foxtail millet quality formation and metabolism, the effects of different Se fertilizers (no application of S1, optimal for Se S2, high Se S3) on yield, quality, and metabolic characteristics of Jingu 21 and Jingu 40 (Se‐rich varieties) were studied. The results showed that S2 treated foxtail millet yield was the highest, the content of fat and starch in Jingu 21 increased, the content of protein decreased, the content of protein in Jingu 40 increased, and the content of starch and fiber decreased. S3 treatment reduced the content of fat and fiber and increased the content of starch. The peak viscosity and breakdown value of Jingu 21 decreased under S2 treatment. Metabolomics analysis revealed that S2 promoted flavonoid accumulation (Taxifolin, Epicatechin, etc.) and inhibition of L‐histidine expression in Jingu 21 by regulating flavonoid and histidine metabolism pathways, leading to reduced protein content, increased fat and starch content, and lower peak viscosity and breakdown value, thereby affecting foxtail millet quality formation. S2 treatment activated the stilbenoid, diarylheptanoid, and gingerol biosynthesis pathways of Jingu40, reducing the synthesis of demethoxycurcumin and chlorogenic acid, increasing protein content and decreasing fat content. S3 treatment activated the tyrosine metabolism pathway of Jingu40, up‐regulated L‐tyrosine and gentisic acid expression, which increased protein content and decreased fat content, thus affecting foxtail millet quality. This study provides a theoretical basis for the safe use of foxtail millet Se fertilizer, high‐quality cultivation, and the development of Se‐enriched functional foods.

## Graphical abstract

S2 treatment regulated the flavonoid and histidine metabolism pathways of Jingu 21, reduced the protein content, increased the fat and starch contents, and decreased the peak viscosity and breakdown value. S2 treatment regulated the Stilbenoid, diarylheptanoid, and gingerol pathway of Jingu 40, and S3 treatment activated the tyrosine metabolic pathway of Jingu 40, increasing protein content and reducing fat content, thereby affecting the quality formation of foxtail millet. This study reveals the metabolic regulatory mechanism of Se fertilizer on the quality formation of foxtail millet.

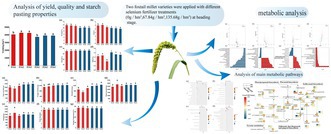

## 1 Introduction

Foxtail millet ( *Setaria italica* L.) originated in China. It has the characteristics of a short growth period, drought resistance, and barrenness resistance. It is rich in protein, dietary fiber, minerals, vitamins, and other nutrients (Chen et al. 2024). Widely cultivated in arid and semi‐arid regions of northern China, foxtail millet plays a significant role in ensuring food production diversity and food security (Zhang et al. 2007). Foxtail millet is rich in selenium (Chen et al. 2024). Selenium (Se) is an essential micronutrient for human health. It is the catalytic center of selenoproteins such as glutathione peroxidase (GSH‐Px) and thioredoxin reductase. It is also an important component of various active enzymes, amino acids, and polysaccharides (Kurokawa and Berry 2013). It has the functions of anti‐oxidation, immune regulation, and gene regulation, and has important application value in the fields of agriculture, environmental protection, and dietary food development (Hasanuzzaman et al. 2020; Schiavon et al. 2017).

Se can regulate the activity of antioxidant enzymes such as glutathione peroxidase (GSH‐Px) to enhance the stress adaptability and disease resistance of plants, increase the Se content in plants, promote plant growth and development, and activate the activity of ATP synthase in plant light response and electron transport chain (Schiavon et al. 2017), increase the content of chlorophyll and carotenoid in leaves, thereby enhancing the utilization of light energy and the absorption of minerals by plants to increase yield (Hu et al. 2003). Optimal Se can enhance the activities of SOD and POD, increase the contents of amino acids, soluble sugar, and flavonoids in tomatoes (Zhu et al. 2018), and reduce the content of NO_3_^−^ by enhancing the activities of enzymes such as glutamine synthase (GS) and glutamate synthase (GOGAT) in the nitrogen metabolism process. It can regulate protein metabolism to increase the protein content of crops (Guo et al. 2023). It can also downregulate the synthesis of phospholipase, reduce the degradation of lipids by phospholipase, and increase fat content (Liang et al. 2020). Wang et al. (2022) demonstrated that Se fertilizer could significantly alter the starch content and starch gelatinization characteristics of buckwheat. Se can also function as a signaling molecule to activate signal transduction pathways in plant cells, thereby inducing the synthesis of beneficial metabolites (phenolic acids, terpenoids, and flavonoids, etc.) (Bandehagh et al. 2023). This process promotes the accumulation of total amino acids and increases vitamin C (Vc) content (Hu et al. 2003).

When Se exceeds a certain threshold, it acts as a pro‐oxidant, leading to necrotic leaf lesions, chloroplast structural damage, impaired branch development, reduced root growth, and may even lead to plant death in severe cases (Prins et al. 2011). Hawrylak‐Nowak (2008) demonstrated that high Se treatment caused excessive phosphorus accumulation in maize stem tissues, disrupted mineral balance, impaired growth and development, and inhibited both chlorophyll synthesis and the tricarboxylic acid (TCA) cycle. These effects resulted in leaf chlorosis, premature senescence, reduced photosynthetic capacity, and ultimately decreased yield (Hasanuzzaman et al. 2022). In addition, excessive Se negatively affects nitrogen metabolism in plants and consequently reduces plant quality. Li, Ji, et al. (2023); Li, Wu, et al. (2023) found that high Se treatment reduced the contents of Vc, soluble sugar, protein, total flavonoids, and phenols in mustard.

Se fertilizer significantly affects foxtail millet growth, development, yield, Se content, and quality. Rasool et al. (2020) demonstrated that exogenous Se significantly increased the ear weight, 1000‐grain weight, and yield of foxtail millet by increasing the level of photosynthetic pigment, reducing membrane damage, reducing malondialdehyde (TBARS) content, promoting the absorption of P, K, Ca, Mg, and other elements, increasing chlorophyll content, and enhancing photosynthetic rate (Guo et al. 2014). Se can also enhance the activity of starch synthases, including sucrose synthase (SuSy), ADP‐glucose pyrophosphorylase (AGPase), and soluble starch synthase (SSS), thereby promoting starch accumulation in foxtail millet (Chen et al. 2024). Mu, Du, Jing, et al. (2017) demonstrated that the heading stage represents a critical period for foxtail millet reproductive growth, and that Se nutrition intervention exhibits a dose‐dependent threshold effect on yield formation and quality development. The application of 67.84 g/hm^2^ Se at the heading stage maximized foxtail millet yield and significantly increased the contents of soluble sugar, lysine, folic acid, fat, protein, and starch in grains (Emam et al. 2014). Mu, Du, Zhang, et al. (2017) also confirmed that foliar application of 67.84 g/hm^2^ sodium selenite at the heading stage represented the optimal Se treatment timing and dosage for foxtail millet. However, the metabolic network mechanisms through which Se fertilizer application at the heading stage regulates foxtail millet quality formation remain unclear. Therefore, this study employed plant physiological analyses and non‐targeted metabolomics (LC–MS) to investigate the effects of optimal Se (67.84 g/hm^2^) and high Se (135.68 g/hm^2^) treatments at the heading stage on foxtail millet's agronomic traits, yield, quality, starch pasting properties, and metabolic characteristics. The research aims to elucidate the regulatory mechanism of Se fertilization on quality formation and metabolism in foxtail millet and provide theoretical support for establishing a safe production system for Se‐enriched foxtail millet and achieving directional quality regulation.

## 2 Materials and Methods

### 2.1 Experimental Overview

The experiment was conducted at the Dingxiang Experimental Base of the Genetic Resources Research Center, Shanxi Agricultural University, in 2021. Meteorological data during the growth period are presented in Table 1. The tested foxtail millet varieties were Jingu 21 (P1) and Se‐rich foxtail millet Jingu 40 (P2) (Han et al. 2024), the experiment was arranged in randomized complete blocks with three replicates, and each plot measured 5 m × 6 m. Na_2_SeO_3_ (Yien Chemical Technology Co. Ltd., Shanghai, China) was used as the Se source. The Se fertilizer application rate included three treatments: control (S1, 0 g/hm^2^), optimal Se (S2, 67.84 g/hm^2^), and high Se (S3, 135.68 g/hm^2^) (Mu, Du, Zhang, et al. 2017). Field management followed local foxtail millet cultivation practices.

**TABLE 1:** Meteorological data of the foxtail millet growth period.

Month	Precipitation (mm)	Temperature (°C)			Relative humidity (%)	Wind speed, mean (ms^−1^)
		Average	Maximum	Minimum		
5	7.0	19.7	36.2	5.6	44.6	1.4
6	7.0	22.9	36.6	11.9	67.3	0.6
7	7.0	25.2	38.5	14.5	79.6	0.1
8	7.0	22.5	35.5	10.9	79.1	0
9	7.0	19.6	33.8	7.7	81.5	0

### 2.2 Determination Indexes and Methods

#### 2.2.1 Agronomic Traits

After plant maturity, 15 plants per treatment were sampled to measure plant height, spike length, stem diameter, and spike weight. Plant height (cm) was determined from the stem base to the apical point using a tape measure. Spike length (cm) was measured from the stem node to the panicle tip with a tape measure. Stem diameter (mm) was recorded at the middle of the third internode of the main stem using vernier calipers. Spike weight (g) was measured with an electronic balance. Thousand‐grain weight (g) was determined after natural air‐drying using an automated seed analyzer.

#### 2.2.2 Yield

The yield was manually harvested at maturity after removing two border rows. Threshed and dried grains from each plot were weighed and recorded in kg/hm^2^.

#### 2.2.3 Protein, Fat, Starch, Fiber Content

The protein content was determined using the Kjeldahl method (Li, Ji, et al. 2023; Li, Wu, et al. 2023). Briefly, 0.5 g of foxtail millet flour was weighed into a digestion tube, followed by the addition of 0.5 g copper sulfate, 4.5 g potassium sulfate, and 10 mL concentrated sulfuric acid. Samples were digested at 420°C for 1 h until a clear green solution was obtained. Automatic determination of digested samples was performed on an automatic Kjeltec nitrogen analyzer (Kjeltec 8400, FOSS, Hillerød, Denmark).

Determination of fat content by Soxhlet extraction (Thiex 2009). A total of 2.0 g of foxtail millet powder was weighed into a filter paper thimble, which was then placed in the extraction tube of a Soxhlet apparatus (ST 255 Soxtec, FOSS, Hillerød, Denmark). Fat extraction was performed using petroleum ether. After extraction, the solvent was evaporated, and the residual fat was dried at 105°C for 1 h, cooled to room temperature in a desiccator, and weighed for fat content calculation.

The starch content was measured by anthrone colorimetry using the starch content kit (No. bc0700, Solarbio, Beijing, China) in accordance with the method of Zhuang et al. (2019).

The fiber content was determined using an automatic fiber extractor (Fibertec 8000, FOSS, Hillerød, Denmark) in accordance with the method of Goudar et al. (2023).

#### 2.2.4 RVA

The water‐balanced sample was ground and dispersed in a mortar, passed through a 100‐mesh sieve, and a constant‐moisture aliquot was weighed for moisture content determination using a moisture analyzer. Based on the measured moisture content, the sample quantity for viscosity analysis was calculated. An exact aliquot (error < 0.05 g) was transferred to aluminum foil, mixed with 25 g of sterile water, and analyzed following Qi et al. (2020) using an RVA‐4 rapid viscoanalyzer (Perten Instruments, Macquarie Park, NSW, Australia) to determine starch pasting properties.

#### 2.2.5 Metabolomics Analysis

The samples were analyzed by Beijing Nuohe Zhiyuan Biotechnology Co. Ltd. (Beijing, China) using a non‐targeted metabolomics approach for metabonomic profiling. Tissue samples (100 mg) ground in liquid nitrogen were transferred to a 1.5‐mL EP tube, followed by the addition of 500 μL of 80% methanol aqueous solution. After vortex mixing and ice‐bath incubation for 5 min, centrifugation was performed at 15,000 × g and 4°C for 20 min. An aliquot of the supernatant was diluted with MS‐grade water to achieve a 53% methanol concentration. The diluted solution was centrifuged again (15,000 × g, 4°C, 20 min), and the resulting supernatant was subjected to LC–MS analysis (Want et al. 2013). The raw mass spectrometry data files (.raw format) were imported into the Compound Discoverer 3.1 platform for peak analysis and compound database matching to obtain metabolite identification and quantification data. Data quality control (QC) was then applied to ensure analytical accuracy and reproducibility.

### 2.3 Statistical Analysis

All data were processed using Excel 2023 and SPSS Statistics 26 (SPSS Institute Inc., Chicago, USA). Variance analysis with multiple comparisons was conducted using the LSD method (*p* < 0.05). Principal component analysis (PCA) and partial least squares‐discriminant analysis (PLS‐DA) were performed to obtain variable importance in projection (VIP) values for each metabolite. Statistical significance (*P*‐value) between groups was determined by t‐tests, and fold change (FC) values were calculated. Differential metabolites were screened with thresholds of *p* < 0.05 and |log_2_FC| > 1. Functional annotation and metabolic pathway analysis were performed using the KEGG database (https://www.genome.jp/kegg/pathway.html). A metabolic pathway was considered enriched when the ratio x/n exceeded y/n, and significantly enriched when *p* < 0.05. All figures were generated using OriginPro 2024 and Adobe Illustrator 2021 software. Data are expressed as mean ± standard deviation (*n* = 3).

## 3 Results and Discussion

### 3.1 Changes in Agronomic Traits of Foxtail Millet Under Se Fertilizer Treatment

Se fertilizer significantly affects foxtail millet agronomic traits (Table 2). The plant height of Jingu 21 and Jingu 40 treated with high Se (S3, 135.68 g/hm^2^) was the highest, which increased by 5.0% and 6%, respectively, compared with the control. The spike length of Jingu 21 and Jingu 40 treated with optimal Se (S2, 67.84 g/hm^2^) increased by 4.6% and 5.7%, respectively, compared with the control. The stem diameter of Jingu 21 and Jingu 40 treated with high Se (S3, 135.68 g/hm^2^) was the thickest. The spike weight of P1S3 and P2S2 increased by 15.5% and 14.3%, respectively, compared with the control. The 1000‐grain weight of Jingu 21 and Jingu 40 treated with optimal Se (S2, 67.84 g/hm^2^) was the heaviest. This may occur because Se reduces electrolyte leakage (EL) and malondialdehyde (TBARS) levels (Rasool et al. 2020), and increases chlorophyll content and photosynthetic rate, thereby mitigating membrane damage and subsequently promoting increases in plant height, panicle weight, and 1000‐grain weight in foxtail millet (Jiang et al. 2024).

**TABLE 2:** Effects of different Se fertilizer application rates on agronomic traits of Jingu 21 and Jingu 40.

Treatment	Plant height/cm	Spike length/cm	Stem thickness/cm	Spike weight/g	Thousand‐grain weight/g
P1S1	156.57 ± 0.72b	24.13 ± 0.35b	6.31 ± 0.05ab	19.37 ± 0.09b	2.99 ± 0.02ab
P1S2	164.37 ± 0.49a	25.23 ± 0.20a	6.35 ± 0.06a	22.15 ± 0.60a	3.06 ± 0.04a
P1S3	164.47 ± 0.33a	23.93 ± 0.03b	6.38 ± 0.03a	22.37 ± 0.18a	2.96 ± 0.04b
P2S1	136.47 ± 0.38b	21.67 ± 0.23a	6.19 ± 0.05a	18.11 ± 0.11b	2.86 ± 0.01ab
P2S2	144.20 ± 0.45a	22.33 ± 0.09a	6.25 ± 0.03a	19.82 ± 0.11a	2.93 ± 0.02a
P2S3	144.60 ± 0.50a	22.07 ± 0.22a	6.27 ± 0.04a	19.69 ± 0.22a	2.84 ± 0.03b
Variety	85.67**	9.07*	60.76**	9.736**	19.448**
Treatment	0.047	1.50	0.018	0.12	2.095
Variety*Treatments	0.07	0.295	0.086	0.079	0.792

*Note:* Different letters in the same column of the same variety showed significant differences between different treatments (*P* < 0.05). In the ANOVA, * and ** indicate variable effect at 0.05, and 0.01 significant levels respectively.

### 3.2 Changes in Foxtail Millet Yield Under Se Fertilizer Treatment

**FIGURE 1:**
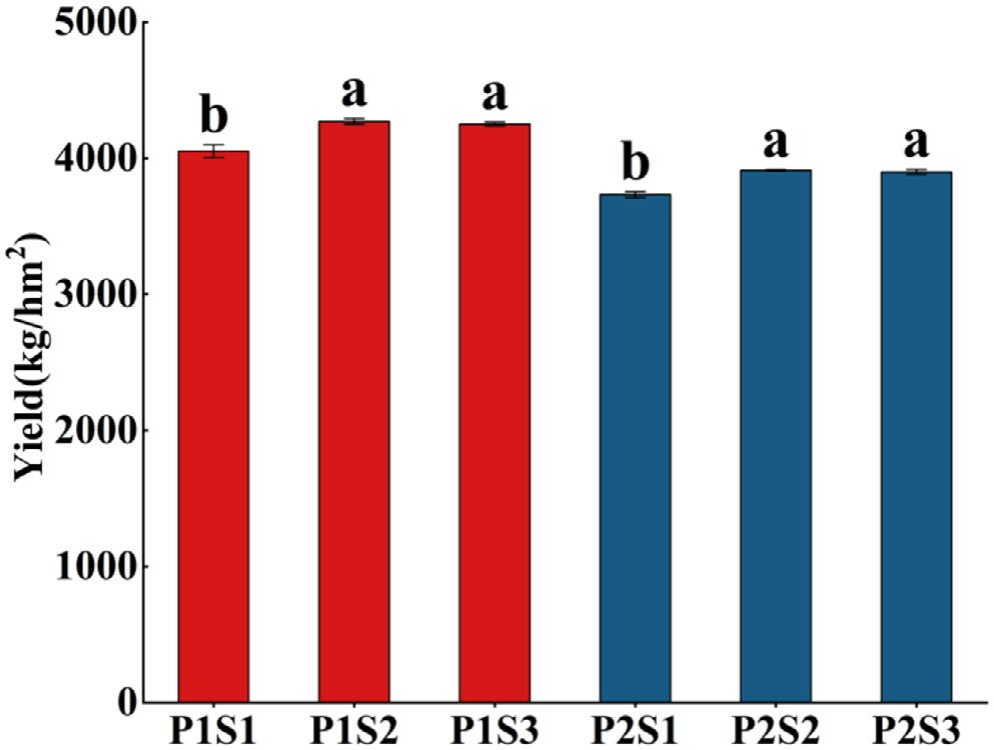
Effects of different Se fertilizer treatments on foxtail millet yield. The vertical bar indicates the standard deviation of three replications. Different letters on the same variety show significant differences at *p* < 0.05.

Se fertilizer significantly increased foxtail millet yield (Figure 1). Jingu 21 and Jingu 40 treated with optimal Se (S2, 67.84 g/hm^2^) demonstrated the highest yields of 4269.65 kg/hm^2^ and 3898.7 kg/hm^2^, respectively, representing 5.3% and 4.8% increases over the control, consistent with findings by Guo et al. (Guo et al. 2014). This may be attributed to Se's ability to activate the activity of ATP synthase in plant photoresponse and the electron transport chain (Schiavon et al. 2017), thereby enhancing the content of proline and chlorophyll, maintaining membrane stability to strengthen plant osmotic protection, improving light energy capture and conversion efficiency, and consequently increasing the photosynthetic rate (Hu et al. 2003). Additionally, Se regulates auxin metabolism and signal transduction, promoting the activity of root meristems and the development of a well‐developed root system, which facilitates better absorption of water and nutrients such as nitrogen, phosphorus, and potassium (Yan, Wu, et al. 2024; Yan, Zhang, et al. 2024). It can also increase the expression level of Se‐sulfur cotransporter in the vascular bundles of foxtail millet, promote the distribution of homogenous compounds to grains during the grain filling period, and specifically accumulate in the embryo of foxtail millet, promoting embryo development and increasing grain fullness (Jiang et al. 2018). These effects collectively improve foxtail millet growth and increase yield.

### 3.3 Changes in Protein, Fat, Starch, and Fiber Content of Foxtail Millet Under Se Fertilizer Treatment

**FIGURE 2:**
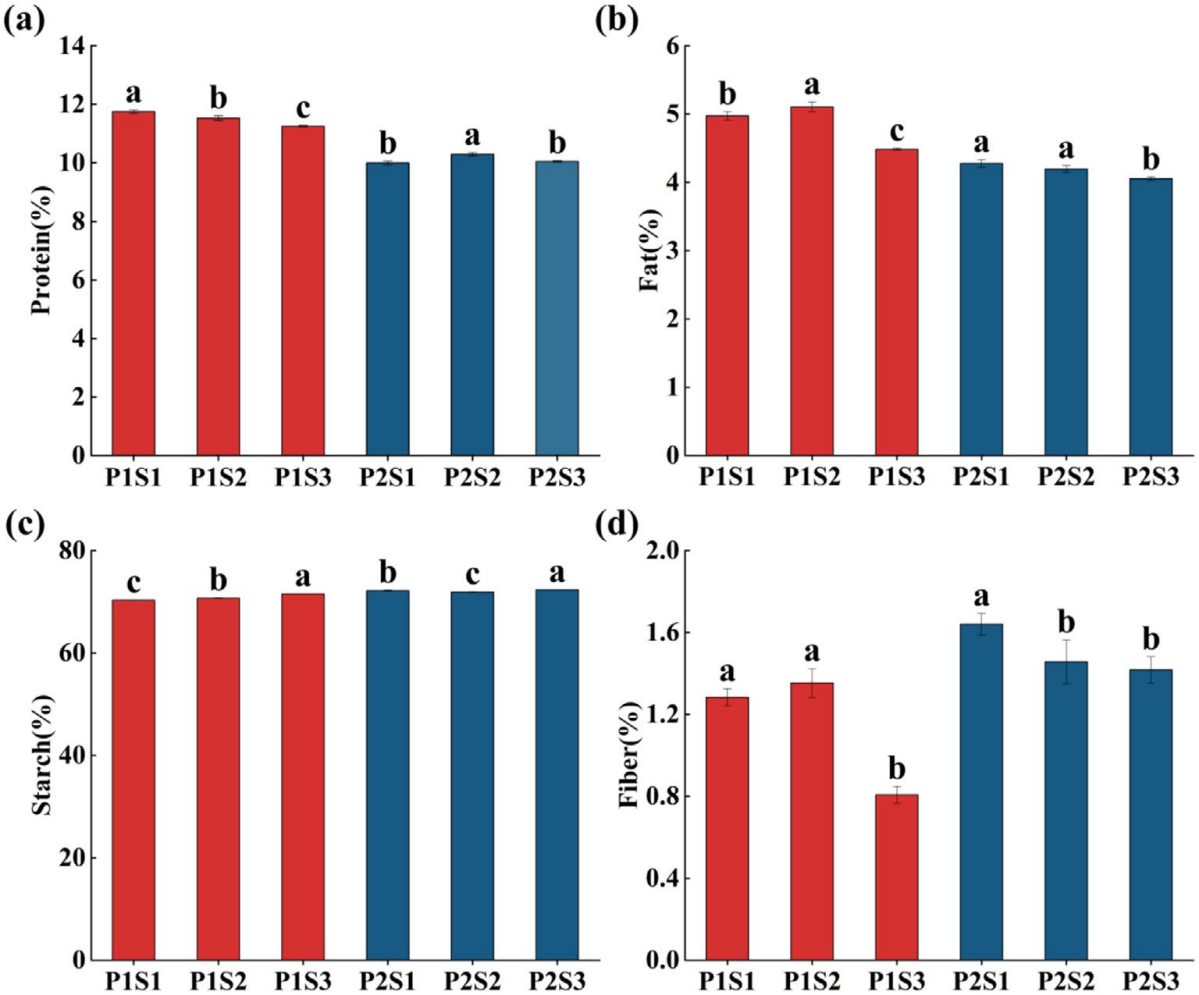
Effects of different Se fertilizer treatments on foxtail millet quality. The vertical bar indicates the standard deviation of three replications. Different letters on the same variety showed significant differences at *p* < 0.05.

Se fertilizer can exert a significant impact on the quality of foxtail millet (Figure 2). Compared to the control, the protein content of Jingu 40 treated with the optimal Se concentration (S2, 67.84 g/hm^2^) increased significantly. This may occur because Se, as a component of antioxidant enzymes, enhances the activity of superoxide dismutase (SOD), peroxidase (POD), and catalase (CAT) (Zhu et al. 2018). It also participates in the nitrogen assimilation pathway to upregulate nitrogen transport genes (OsNRT1.1B), increasing nitrogen absorption efficiency (Zhang and Chu 2022). Simultaneously, it activates glutamine synthase (GS) and glutamate dehydrogenase (GDH), promoting the conversion of nitrogen to amino acids (Guo et al. 2023), ultimately increasing protein content. The protein content of Jingu 21 decreased significantly after high Se treatment (S3, 135.68 g/hm^2^). When the amount of Se fertilizer exceeds the appropriate level, it disturbs normal amino acid metabolism and affects sulfur metabolism, resulting in the replacement of sulfur‐containing amino acids by selenoamino acids, which destroys protein structure (Malagoli et al. 2015), thereby reducing protein content accumulation. The optimal Se treatment (S2, 67.84 g/hm^2^) increased the fat content of Jingu 21, which was consistent with the findings of Mu, Du, Zhang, et al. (2017) in foxtail millet. This may be attributed to the fact that an appropriate amount of Se fertilizer can inhibit the synthesis of phospholipase (Liang et al. 2020). Furthermore, it can reduce the oxidative damage caused by reactive oxygen species (ROS) to acyl‐ACP thioesterase and maintain the redox homeostasis of the fatty acid extension cycle (Guo et al. 2023), thereby promoting the accumulation of fat content. The fat content of Jingu 21 and Jingu 40 treated with high Se (S3, 135.68 g/hm^2^) decreased significantly compared with the control. Excessive Se application interferes with mitochondrial electron transport chain function, increases electron leakage, and consequently generates substantial reactive oxygen species (ROS), including superoxide anions. These ROS induce lipid peroxidation while inhibiting lipoxygenase (LOX) activity. The reduced proportion of unsaturated fatty acids in cell membranes becomes more susceptible to oxidation (Foyer and Noctor 2011), ultimately leading to decreased fat content. In this study, the optimal Se treatment (S2, 67.84 g/hm^2^) significantly increased the starch content of Jingu 21, while the high Se treatment (S3, 135.68 g/hm^2^) simultaneously enhanced starch content in both Jingu 21 and Jingu 40. These findings align with the results reported by Emam et al. (2014). This may occur because Se fertilizer enhances the activity of AGPase, SuSy, and SSS, promotes the conversion of sucrose to ADPG, and increases the starch synthesis rate (Chen et al. 2024). Concurrently, it stimulates photosynthetic pigment synthesis, improves photosynthetic efficiency, and provides abundant carbon sources for starch synthesis (Wang et al. 2022), ultimately elevating the starch content in foxtail millet.

### 3.4 Changes in Foxtail Millet RVA Under Se Fertilizer Treatment

**FIGURE 3:**
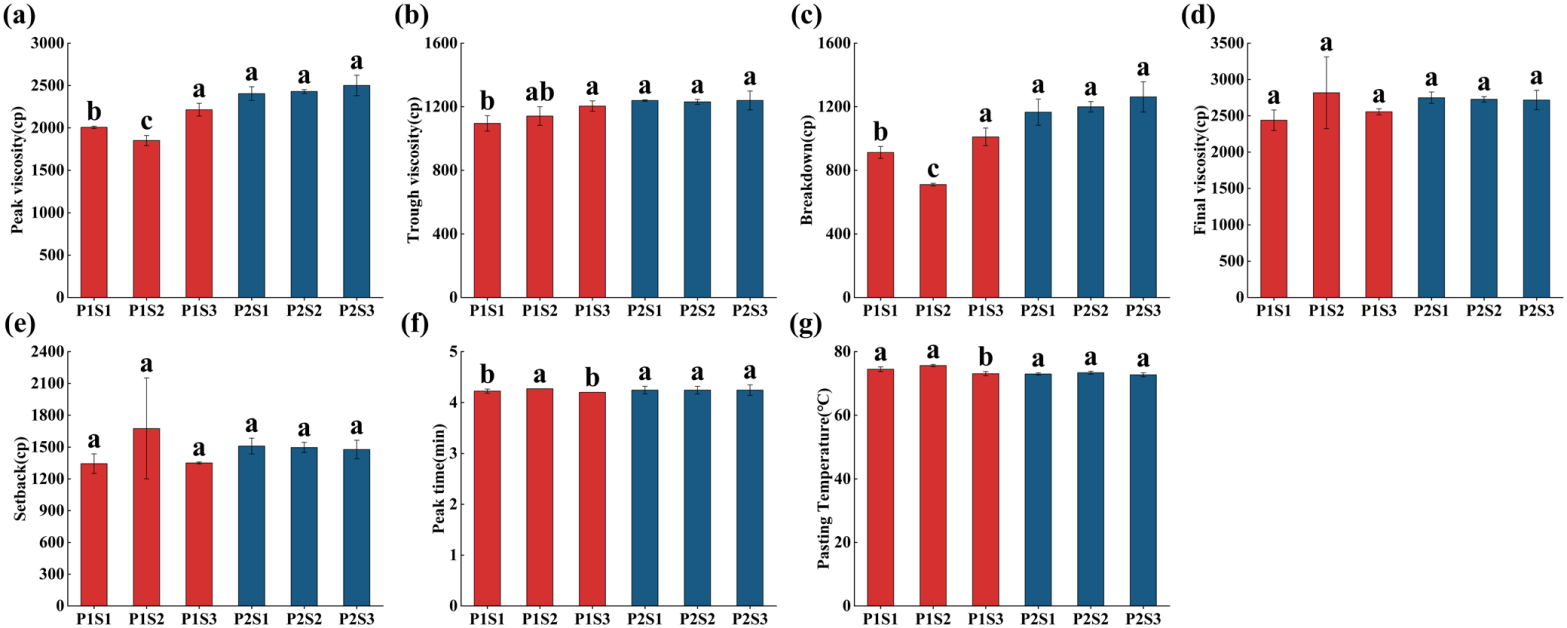
Effect of different Se fertilizer treatments on foxtail millet RVA. The vertical bar indicates the standard deviation of three replications. Different letters on the same variety showed significant differences at *p* < 0.05.

Se fertilizer can exert a significant impact on the RVA of foxtail millet (Figure 3). Compared to the control, the optimal Se treatment (S2, 67.84 g/hm^2^) reduced the peak viscosity and breakdown value of Jingu 21, which was consistent with the results of Wang et al. (2022). The optimal Se treatment (S2, 67.84 g/hm^2^) increased the fat content of Jingu 21 (Figure 2b). Lipids and linear starch molecules (amylose) formed insoluble complexes with single‐helix configurations through hydrophobic interactions. The steric hindrance effect of these complexes inhibited starch granule expansion during thermal processing, thereby reducing peak viscosity and breakdown value (Song and Jane 2000). Conversely, high Se treatment (S3, 135.68 g/hm^2^) increased both peak viscosity and breakdown value in Jingu 21. This might be because Se can enhance the activities of β and total amylase, invertase, and sucrose synthase, and alter the activities of enzymes related to sucrose and starch metabolism (Kaur et al. 2018). As a coenzyme or cofactor, it enhances the activity of starch hydrolases such as α‐amylase and promotes the degradation of starch molecules (Wang et al. 2022). Meanwhile, the increase in SSS activity can catalyze the extension of α‐1, 4‐glycosidic bonds, transferring the glucose groups in ADPG to the non‐reducing ends of amylopectin. This increases the content of amylopectin and long‐chain amylopectin (Tian et al. 2009; Yang et al. 2016). Moreover, Se‐rich crops have a high thiol content, a low protein content (Figure 2a), few disulfide bonds, and a low degree of interprotein cross‐linking (Martin and Fitzgerald 2002), further reducing the extent to which starch is tightly encapsulated by proteins. This makes it easier for water molecules to combine with starch, thereby increasing the peak viscosity and breakdown value (Wang et al. 2021). Starch with high peak viscosity and breakdown value can form a dense gel network with better taste and texture (Li et al. 2012).

### 3.5 Metabolomics Analysis

#### 3.5.1 Quality Control of Metabolomics Data

**FIGURE 4:**
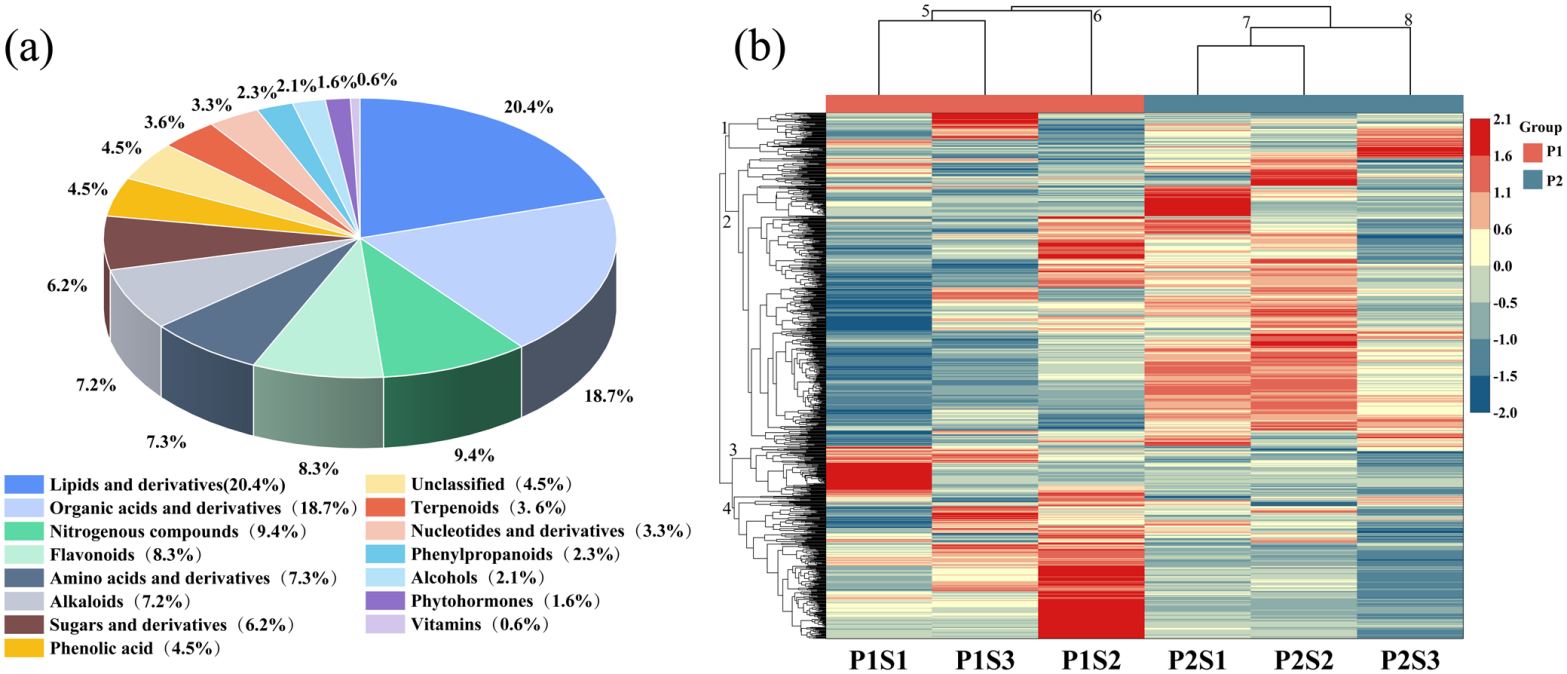
(a) Foxtail millet metabolic category analysis. (b) Foxtail millet metabolic clustering hierarchical clustering analysis (HCA).

In this study, the Pearson correlation coefficient was employed to assess data homogeneity within replicate groups. When |r| approaches 1, it indicates higher data consistency (Figure S1). Under the LC–MS/MS platform, non‐targeted metabolomics techniques were used to study the changes in metabolites in two different genotypes of foxtail millet. A total of 822 metabolites were identified across all samples, classified into 15 categories (Figure 4a). The predominant categories includedlipids and derivatives (168 metabolites, 20.4%), organic acids and derivatives (154 metabolites, 18.7%), nitrogen‐containing compounds (77 metabolites, 9.4%), flavonoids (68 metabolites, 8.3%), amino acids and derivatives (60 metabolites, 7.3%), and sugars and derivatives (51 metabolites, 6.2%). The content of metabolites was normalized for hierarchical clustering analysis (HCA). The content of metabolites was normalized to construct a hierarchical clustering heat map. The results revealed that the distribution of related metabolites in two foxtail millet varieties and different Se fertilizer application rates was divided into four categories (Figure 4b). Clusters 1 and 2 showed that Jingu 21 had the highest metabolite abundance; clusters 3 and 4 showed that Jingu 40 had the highest metabolite abundance. The changes in metabolite contents in Jingu 21 and Jingu 40 indicated that they were related to the varieties of foxtail millet. Clusters 5, 6, 7, and 8 demonstrated that metabolite content variations in both Jingu 21 and Jingu 40 were associated with different Se fertilizer application rates (Figure 4b).

PCA analysis of the metabolites of foxtail millet under different Se fertilizer application rates (Figure S2) demonstrated that the cumulative contribution of the first two principal components (PC1 was 31.61%, PC2 was 18.59%) was 50.2%. Significant differences in metabolites were observed between treatment groups, though individual replicate samples within each group showed substantial dispersion, indicating discrete data points. Based on this pattern, partial least squares‐discriminant analysis (PLS‐DA) was performed with incorporated grouping variables (Figure S3). In all PLS‐DA score plots, obvious separation trends were observed between sample groups. R^2^Y represents the explained variance of the model, while Q^2^Y reflects its predictive power. In all PLS‐DA score plots, R^2^Y values were close to 1 and consistently higher than Q^2^Y, indicating robust model performance. The Q2 values of all the original models in the model ranking verification diagram (Figure S4) were much larger than those of the random model, and the Q2 regression line and Y‐axis intercept were less than 0, indicating that the samples detected in this study had good repeatability and stability, which could be used for subsequent differential metabolite analysis.

#### 3.5.2 Screening of Differential Metabolites

**FIGURE 5:**
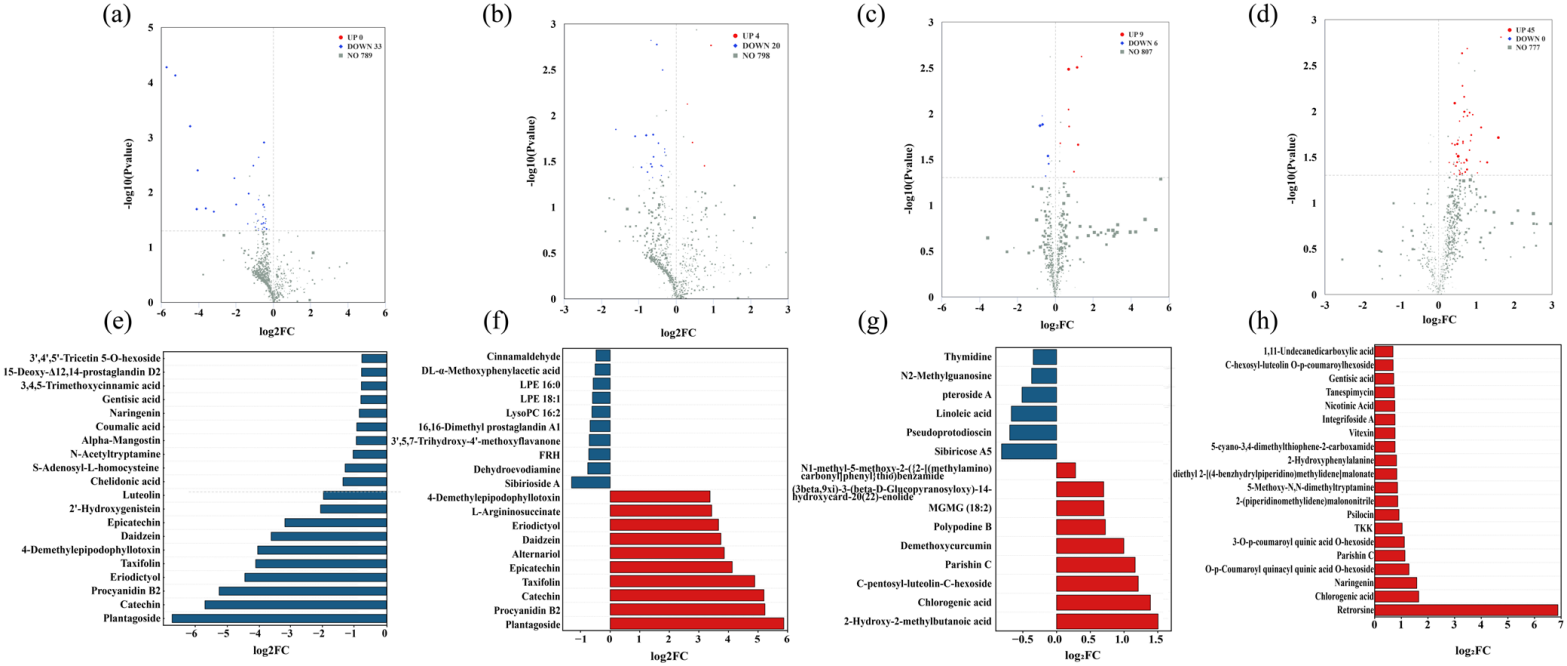
DAMs up‐ and down‐regulation in various treatment groups. (a–d) P1S1 vs. P1S2, P1S1 vs. P1S3, P2S1 vs. P2S2, P2S1 vs. P2S3, showing volcanic maps of up‐regulated and down‐regulated metabolites. (e–h) P1S1 vs. P1S2, P1S1 vs. P1S3, P2S1 vs. P2S2, P2S1 vs. P2S3, showing up‐regulation and down‐regulation of the top 20 differential metabolites.

In order to identify the most important metabolites in Jingu 21 and Jingu 40 under different Se fertilizer treatments, this study combined the PLS‐DA model to calculate the important projection value (VIP) and variance analysis between groups. The threshold values were set as VIP > 1.0, |log_2_FC| > 1, and *p* < 0.05, and the selected metabolites were defined as differential metabolites (DAMs). There were 33 differential metabolites screened between P1S1 and P1S2 (0 up‐regulated, 33 down‐regulated), and 24 differential metabolites screened between P1S1 and P1S3 (4 up‐regulated, 20 down‐regulated). There were 15 differential metabolites screened out in P2S1 and P2S2 (9 up‐regulated and 6 down‐regulated), and 45 differential metabolites screened out in P2S1 and P2S3 (45 up‐regulated and 0 down‐regulated). According to the order of log2FC from large to small, the top 10 differential metabolites up‐regulated and down‐regulated in P1S1 vs. P1S2, P1S1 vs. P1S3, P2S1 vs. P2S2, and P2S1 vs. P2S3 were labeled (Figure 5e–h). The top 20 differential metabolites with the largest down‐regulation in P1S1 vs. P1S2 were mainly flavonoids, organic acids and derivatives, and lipids and derivatives. The top 10 differential metabolites with the largest down‐regulation in P1S1 vs. P1S3 were mainly lipids and derivatives, flavonoids and sugars and derivatives, and the compounds with the largest up‐regulation were mainly flavonoids, amino acids, and derivatives. The most down‐regulated differential metabolites in P2S1 vs. P2S2 were mainly lipids and derivatives, sugars and derivatives, and nucleotides and derivatives, and the most up‐regulated compounds were flavonoids, phenolic acids, yellow organic acids and derivatives, and terpenoids. The most up‐regulated compounds in P2S1 vs. P2S3 were mainly alkaloids, phenolic acids, flavonoids, sugars, and derivatives.

#### 3.5.3 KEGG Enrichment Analysis of DAMs

**FIGURE 6:**
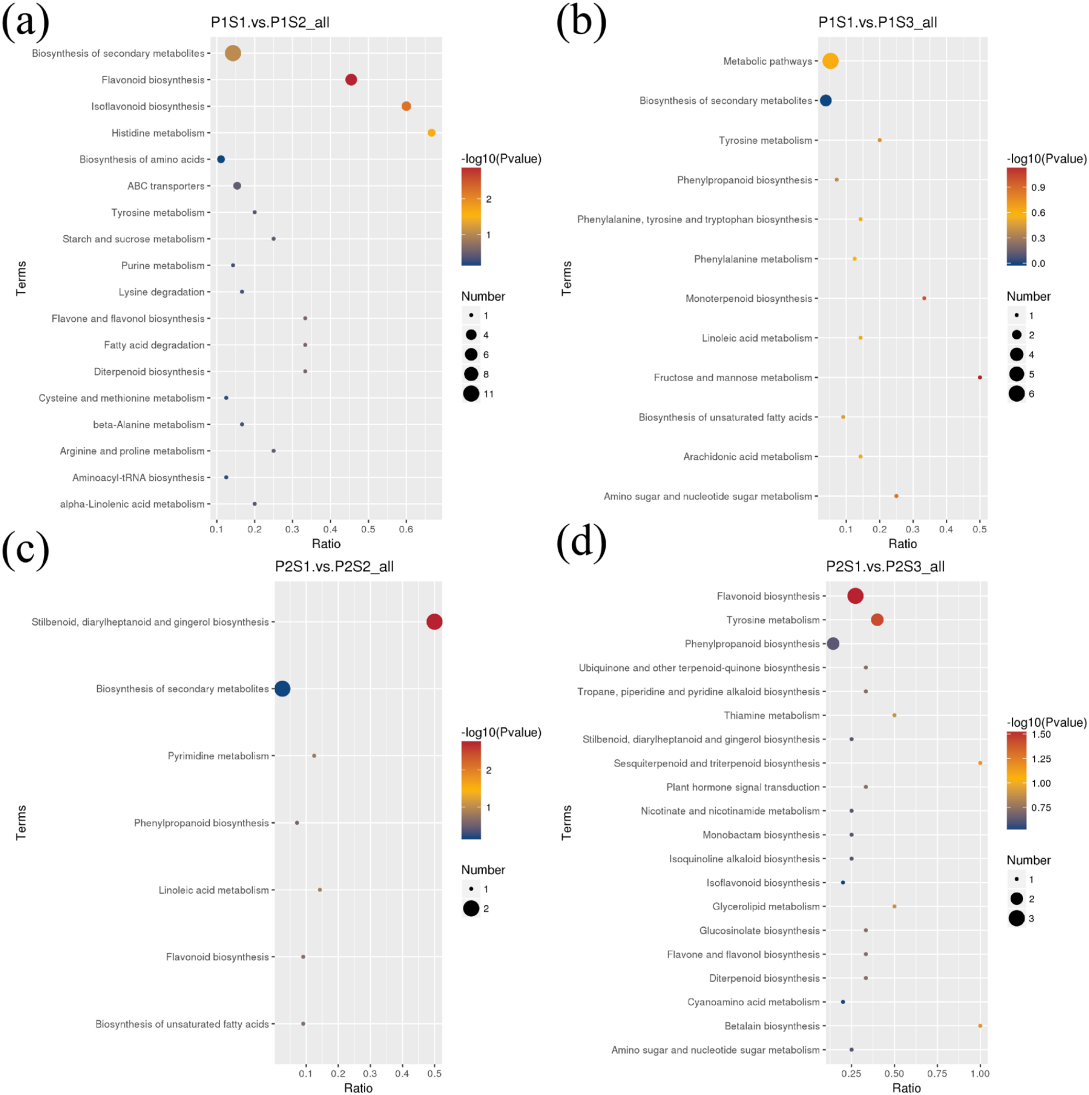
Pathway analysis of differential accumulation metabolites. (a–d): KEGG pathway enrichment of differential metabolites between groups (P1S1 vs. P1S2, P1S1 vs. P1S3, P2S1 vs. P2S2, P2S1 vs. P2S3). Each bubble in the figure represents a metabolic pathway. The *X* axis is the enrichment ratio. The *Y* axis is the path name. The bubble size represents the number of DAMs involved, and the bubble color represents the degree of pathway enrichment.

According to the KEGG database, pathway enrichment analysis was performed on the differential metabolites of foxtail millet to facilitate an overview of the changes in metabolic regulation of foxtail millet after Se fertilizer treatment. In this study, the differential metabolites of each group were annotated and classified into different pathways. Among them, the differential metabolites of P1S1 vs. P1S2, P1S1 vs. P1S3, P2S1 vs. P2S2, and P2S1 vs. P2S3 involved 18, 12, 7, and 29 pathways (Figure 6a–d). In P1S1 vs. P1S2, the flavonoid biosynthesis, isoflavonoid biosynthesis, and histidine metabolism pathways were significantly enriched, and the levels of Taxifolin, Naringenin, Epicatechin, Eriodictyol, and Luteolin in the flavonoid biosynthesis pathway were increased. 2′‐Hydroxygenistein, Naringenin, and Daidzein were down‐regulated in the isoflavonoid biosynthesis pathway. There was no significant enrichment pathway in P1S1 vs. P1S3. In P2S1 vs. P2S2, stilbenoid, diarylheptanoid, and gingerol biosynthesis pathways were significantly enriched, and Chlorogenic acid (CGA) and Demethoxycurcumin (DMC) were up‐regulated in this pathway. In P2S1 vs. P2S3, the flavonoid biosynthesis and tyrosine metabolism pathways were significantly enriched. Naringenin, CGA, and Vitexin were up‐regulated in flavonoid biosynthesis, and Gentisic acid and L‐Tyrosine were also up‐regulated in the tyrosine metabolism pathway (Figure 6d).

### 3.6 Correlation Analysis

Pearson correlation analysis was performed on the DAMs in the significant enrichment pathway of Jingu 21 and Jingu 40 after different Se fertilizer treatments and the physiological indices of foxtail millet. In Jingu 21, protein was negatively correlated with Luteolin and L‐Histidine, significantly negatively correlated with Taxifolin and Naringenin, and extremely significantly negatively correlated with Epicatechin and Eriodictyol. Fat was positively correlated with Luteolin, Taxifolin, Naringenin, Epicatechin, and Eriodictyol, and significantly positively correlated with L‐Histidine. Starch was significantly positively correlated with Luteolin, Naringenin, and L‐Histidine, and was significantly positively correlated with Taxifolin, Epicatechin, and Eriodictyol. The peak viscosity was negatively correlated with Naringenin, Epicatechin, and Eriodictyol, significantly negatively correlated with Taxifolin and L‐Histidine, and significantly negatively correlated with Luteolin. The breakdown value was negatively correlated with DAMs. Both setbacks and pasting temperature were positively correlated with DAMs. In Jingu 40, protein and starch were negatively correlated with L‐Tyrosine and Gentisic acid, and fat was positively correlated with L‐Tyrosine and Gentisic acid.

### 3.7 Critical Metabolic Pathway Analysis

**FIGURE 7:**
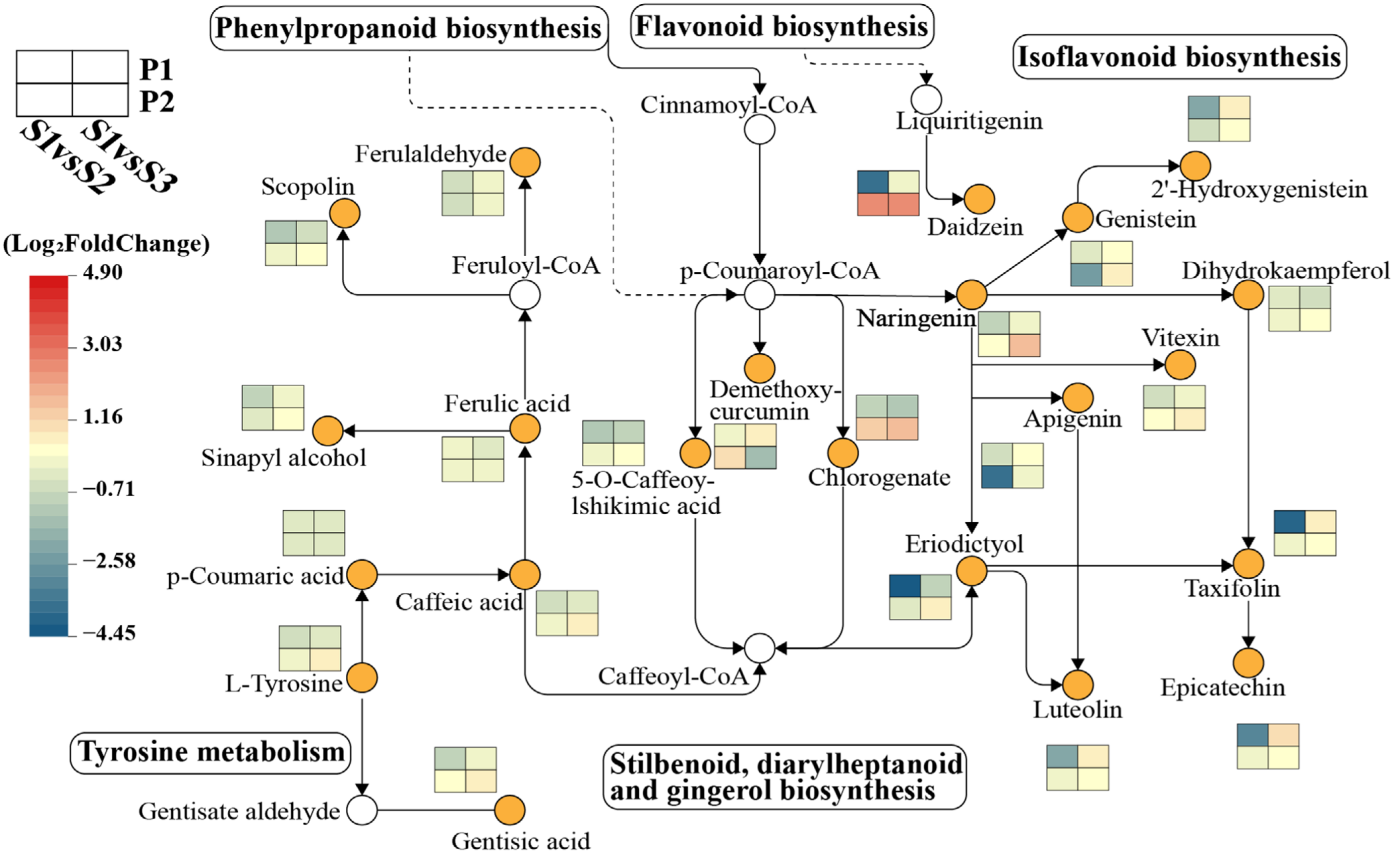
Orange circles represent differential metabolites with changed content, and white circles represent metabolites with unchanged content. The first row of the rectangle is represented from left to right (P1S1 vs. P1S2, P1S1 vs. P1S3), and the second row is represented from left to right (P2S1 vs. P2S2, P2S1 vs. P2S3). The color of the rectangle indicates that the metabolites are regulated under Se treatment, as described by the scale.

**FIGURE 8:**
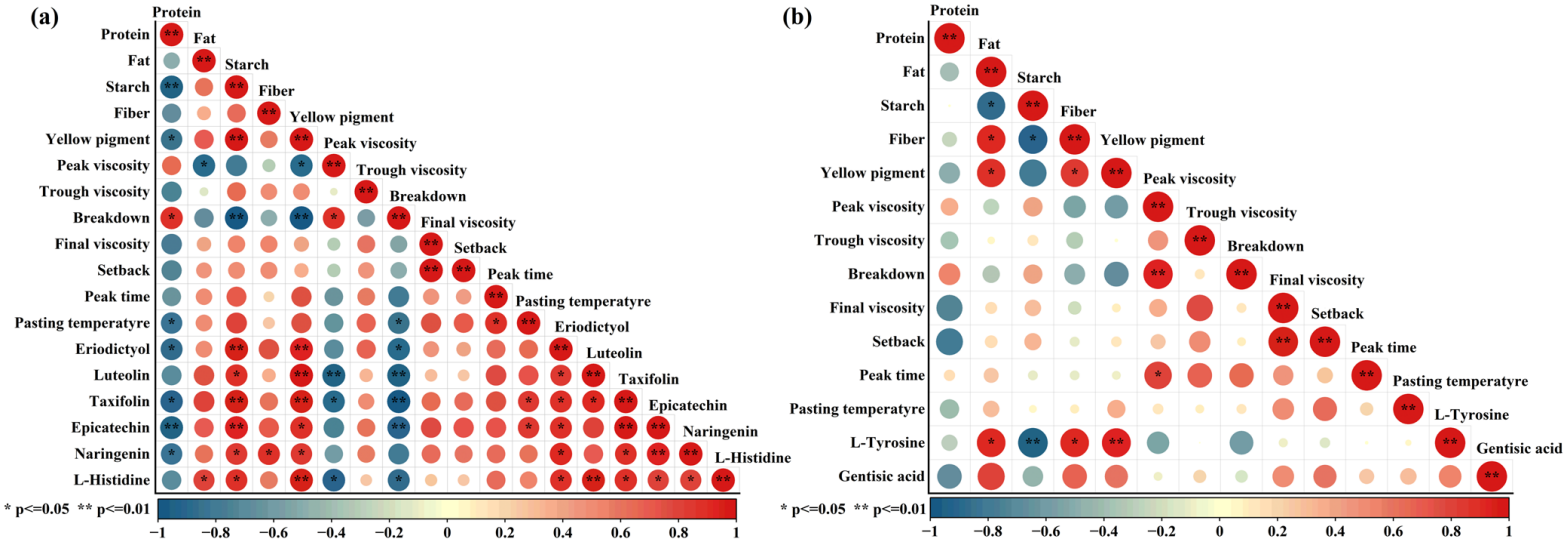
Correlation analysis of physiological indexes and differential metabolites of Jingu 21 and Jingu 40 treated with different Se fertilizers: (a) Jingu 21; (b) Jingu 40.

According to the KEGG pathway enrichment analysis of differential metabolites in each comparison group, we found that DAMs were mainly significantly enriched in Flavonoid biosynthesis, Histidine metabolism, Stilbenoid, diarylheptanoid, and gingerol biosynthesis, and Tyrosine metabolism pathways (Figure 7). Se fertilizer can promote the upregulation of NAD‐dependent heteroisomerase, REDOX zinc‐binding dehydrogenase, and acyl‐CoA synthetase expressions in Phenylpropanoid biosynthesis (Zhu et al. 2018), and upregulate CYP73A enzyme‐related genes. Promote the conversion of Caffeic acid to p‐coumaric acid under the action of 4‐coumaric acid‐coenzyme A ligase (Liu et al. 2024), which is a key precursor for the synthesis of flavonoids. It can increase the synthesis of flavonoids such as Taxifolin, Naringenin, Epicatechin, Eriodictyol, and Luteolin. The accumulation of flavonoids will competitively reduce the precursor substances for the synthesis of Demethoxycurcumin (DMC) and Chlorogenic acid (CGA), resulting in a decrease in the contents of DMC and CGA (Yang et al. 2023). Both DMC and CGA have antioxidant functions. The decrease in the contents of DMC and CGA may release more phenylalanine into primary metabolism (Yang et al. 2024). Phenylalanine is a direct precursor for protein synthesis, thereby promoting the increase of protein content in Jingu 40 (Figure 2a). The synthesis of flavonoids requires the consumption of a large amount of phenylalanine and malonyl‐CoA, resulting in the shift of nitrogen sources to secondary metabolism and promoting the increase of soluble sugar (Ibrahim et al. 2011). The accumulation of soluble sugar is related to the regulation of starch and sucrose metabolism by Se fertilizer and the upregulation of gene expression, such as MD02G1100500 and MD06G1237200 (Liu et al. 2024). Soluble sugar activates the TOR signaling pathway (Lando et al. 2024), inhibits the expression of nitrogen absorption‐related genes (nitrate transporter NRT, nitrite reductase NIR, etc.), reduces nitrogen assimilation into amino acids, and limits protein synthesis (Guo et al. 2023), thereby reducing the protein content of Jingu 21 (Figure 2a). The decrease in protein content will lead to a decrease in disulfide bonds, the weakening of cross‐linking between protein molecules, and the inhibition of adhesion, thus reducing the dense binding degree of starch and protein, limiting the interaction between water molecules and starch (Wang et al. 2021). The protein network structure fails, and the expansion of starch granules is inhibited, resulting in a decrease in the peak viscosity and breakdown value of Jingu 21. Low peak viscosity and breakdown values are more likely to meet the processing quality requirements of millet in the production of low‐hardness noodles (Hara et al. 2009). Correlation analysis also showed that flavonoid metabolites such as taxifolin and epicatechin were negatively correlated with peak viscosity and breakdown value (Figure 8). Taxifolin and epicatechin are antioxidants, and the increase in their content more effectively removes reactive oxygen species (ROS), reduces the damage of lipid peroxidation to fat synthase (FAS and ACC) (Chobot et al. 2016), and promotes the increase of fat content in Jingu 21 (Figure 2b). The increase of flavonoids may also affect the tricarboxylic acid cycle (TCA cycle), increase ATP content (Treutter 2005), participate in the hydrolysis and sugar metabolism processes of starch, regulate the activity of starch synthase, and promote the increase of starch content in Jingu 21 (Figure 2c) (Wang et al. 2023). These results suggest that optimal Se increases flavonoids by regulating flavonoid metabolism in Jingu 21, increases antioxidant capacity, increases fat content, inhibits nitrogen metabolism expression, and reduces protein content as well as peak viscosity and breakdown value. Optimal Se regulates the stilbenoid, diarylheptanoid, and gingerol biosynthesis pathway of Jingu 40, promotes the increase of protein content by reducing the biosynthesis of DMC and CGA, reduces fat content, and affects the formation of foxtail millet quality.

The L‐Histidine of Jingu 21 was significantly enriched in the histidine metabolic pathway under the optimal Se treatment (S2, 67.84 g/hm^2^). Histidine synthesis depends on PRPP as a precursor. Se may competitively inhibit the activity of phosphoribose pyrophosphate synthase (PRPP), resulting in blocked histidine synthesis (Hove‐Jensen et al. 2017). Histidine is one of the important amino acids for protein synthesis (Galili et al. 2016). Down‐regulation of its expression may further limit protein synthesis (Figure 2a). Decreased PRPP activity may affect nucleotide metabolism (Vavassori et al. 2005). Nucleotide metabolism (ATP/ADP ratio) promotes the production of AGPase, and AGPase cooperates with sucrose synthase (SuSy) to produce adenosine diphosphate glucose (ADPG), the substrate of starch synthesis (Yan, Wu, et al. 2024; Yan, Zhang, et al. 2024). The increase in its content can promote the accumulation of foxtail millet starch content (Figure 2c). The DAMs of Jingu 40 treated with high Se (S3, 135.68 g/hm^2^) were significantly enriched in the tyrosine metabolic pathway, and L‐Tyrosine was upregulated. Se can directly participate in protein metabolism by substituting sulfhydryl groups (‐SH) or integrating into the active centers of enzymes, replacing sulfur in sulfhydryl groups to form seleninated amino acids (such as selenocysteine) (Hatfield et al. 2014), and regulating the activities of phenylalanine hydroxylase (PAH) or tyrosine aminotransferase (TAT). This promotes the conversion of phenylalanine to tyrosine (Xi et al. 2019). L‐Tyrosine is not only the basic structural unit of protein but also the synthetic precursor of plant‐specific metabolites (PSM) (Muttucumaru et al. 2014). When ribosomes translate mRNA to synthesize proteins, L‐Tyrosine can successfully complete the extension of the polypeptide chain, thereby increasing the synthesis of some key enzyme proteins and structural proteins (Hwang et al. 2025), thereby increasing the total protein content (Figure 2a). In this study, optimal Se down‐regulated L‐histidine by regulating the histidine metabolic pathway of Jingu 21, reduced protein content, and promoted AGPase production to increase starch content. High Se regulated the tyrosine metabolism pathway of Jingu 40, accumulated more antioxidants, increased protein content, and reduced fat content, thereby improving the grain quality of Jingu 40.

## 4 Conclusion

By studying the effects of Se fertilizer treatment on the agronomic traits, yield, quality, and metabolic characteristics of Jingu 21 and Jingu 40, we found that optimal Se (S2, 67.84 g/hm^2^) treatment maximized the yield of Jingu 21 and Jingu 40, increased the fat and starch content of Jingu 21, and decreased the protein content. Under optimal Se (S2, 67.84 g/hm^2^) treatment, the protein content of Jingu 40 increased, while the contents of starch and fiber decreased. High Se (S3, 135.68 g/hm^2^) treatment reduced the fat and fiber content of the two varieties but increased the starch content. Se (S2, 67.84 g/hm^2^) treatment reduced the peak viscosity and breakdown value of Jingu 21. Metabolomics analysis showed that optimal Se (S2, 67.84 g/hm^2^) treatment promoted the accumulation of flavonoids (Taxifolin, epicatechin, etc.) in Jingu 21 by regulating flavonoid metabolism and histidine metabolic pathways, improved antioxidant capacity, and promoted carbon and nitrogen metabolism to reduce protein synthesis. The optimal Se (S2, 67.84 g/hm^2^) treatment regulated the Stilbenoid, diarylheptanoid, and gingerol biosynthesis pathways of Jingu 40, promoting the increase of protein content by reducing the contents of DMC and CGA. High Se (S3, 135.68 g/hm^2^) treatment activated the tyrosine metabolic pathway of Jingu 40, up‐regulated the expression of L‐tyrosine and gentisic acid, and increased the protein content, thus affecting the quality formation of foxtail millet. This study reveals the metabolic regulatory mechanism of Se fertilizer on the quality formation of foxtail millet, which is conducive to further exploring the theory of physiological metabolic regulation of crops under Se fertilizer treatment and provides a theoretical basis for the safe use of Se fertilizer for foxtail millet and high‐yield and high‐quality cultivation.

## Author Contributions

**Shu Wang:** conceptualization (equal), data curation (lead), formal analysis (lead), methodology (equal), project administration (lead), resources (lead), supervision (lead), visualization (lead), writing‐original draft (lead). **Jiao Mao:** conceptualization (equal), investigation (equal), methodology (equal), writing‐review and editing (equal). **Yuanmeng Xu:** investigation (equal), methodology (supporting), writing‐review and editing (equal). **Sichen Liu:** writing, review, and editing (equal). **Zhijun Qiao:** conceptualization (equal), supervision (equal), writing‐review and editing (equal). **Xiaoning Cao:** methodology (equal), visualization (equal), writing‐original draft (supporting), funding acquisition.

## Ethics Statement

The authors have nothing to report.

## Consent

The authors have nothing to report.

## Conflicts of Interest

The authors declare no conflicts of interest.

## Supporting information


Figure S1.
Figure S2.
Figure S3.
Figure S4.

## Data Availability

For access to the data utilized in this research, readers are encouraged to reach out to the corresponding author.
