# Supplementary material for: Optimal Selenium Fertilizer Affects the Formation of Foxtail Millet ( Setaria italica L.) Quality by Regulating Flavonoid Metabolism and Amino Acid Metabolism
Source: Food Sci Nutr. 2025 May 30;13(6):e70362. doi: 10.1002/fsn3.70362 (PMC12124234; doi:10.1002/fsn3.70362)
Supplement: Supplementary file 1 — Figure S1. Figure S2. Figure S3. Figure S4. [file FSN3-13-e70362-s001.docx]

**Supplementary figures**

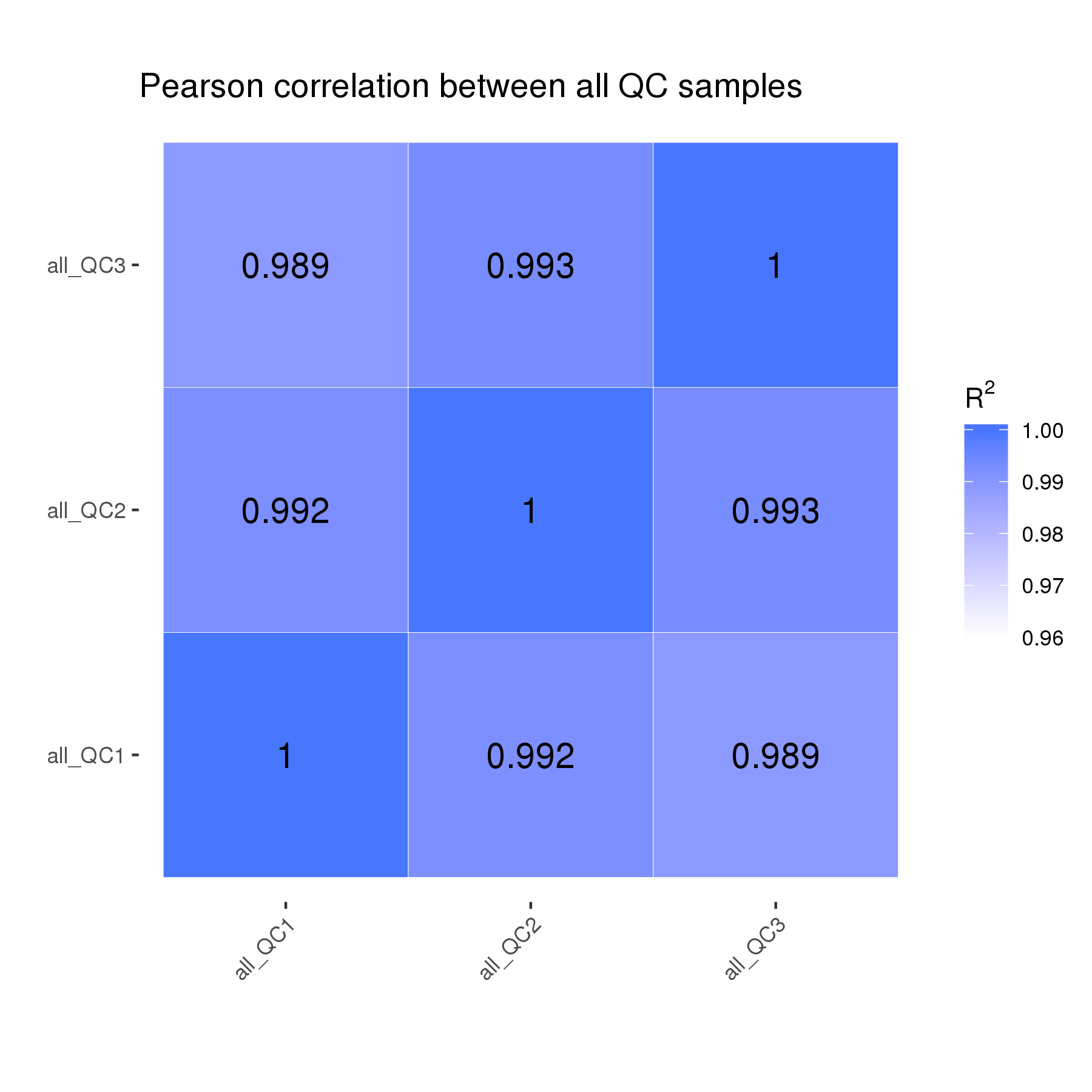


Fig. S1. QC sample correlation analysis. The higher the correlation (R2 is closer to 1), the better the stability of the entire detection process and the higher the data quality.


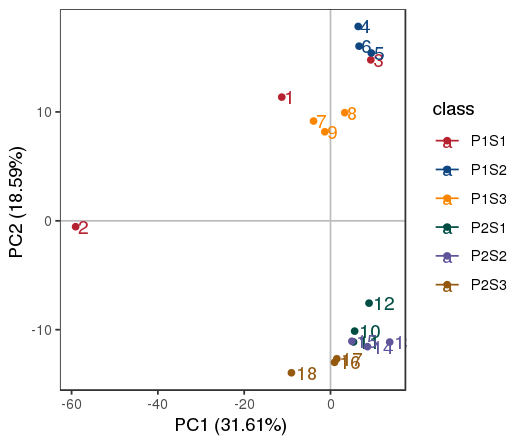


Fig. S2. PCA analysis of foxtail millet metabolites. The colored dots in the diagram represent each sample and the numbers are the sample numbers.


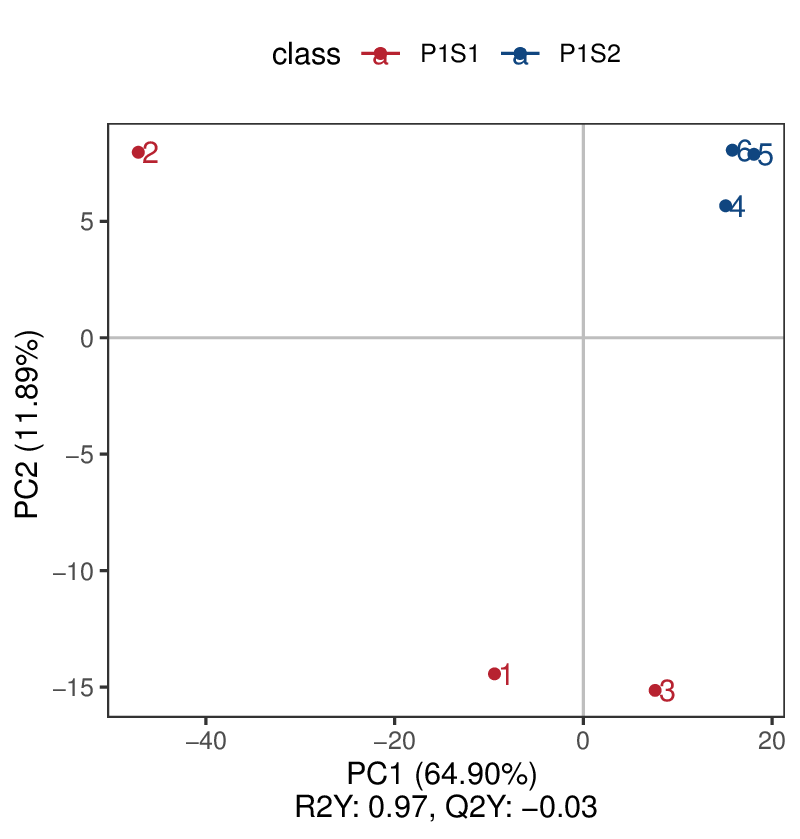

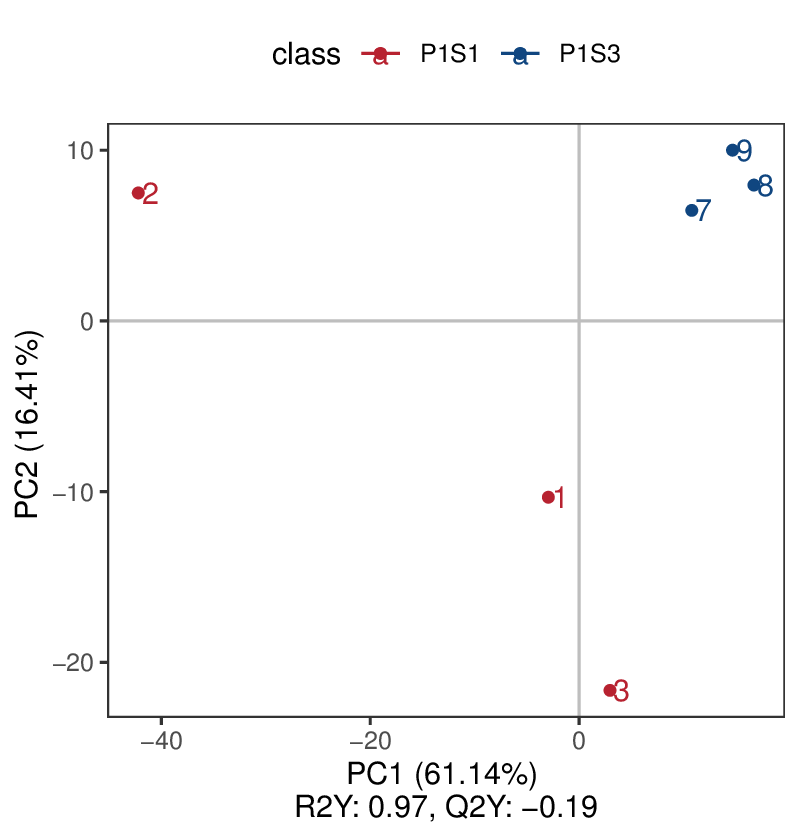


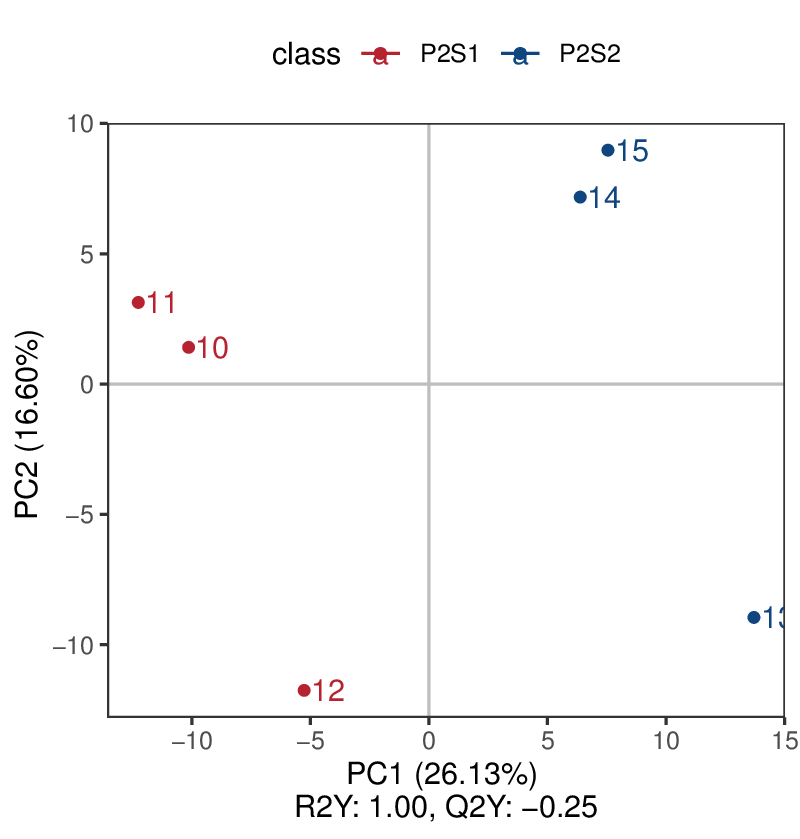

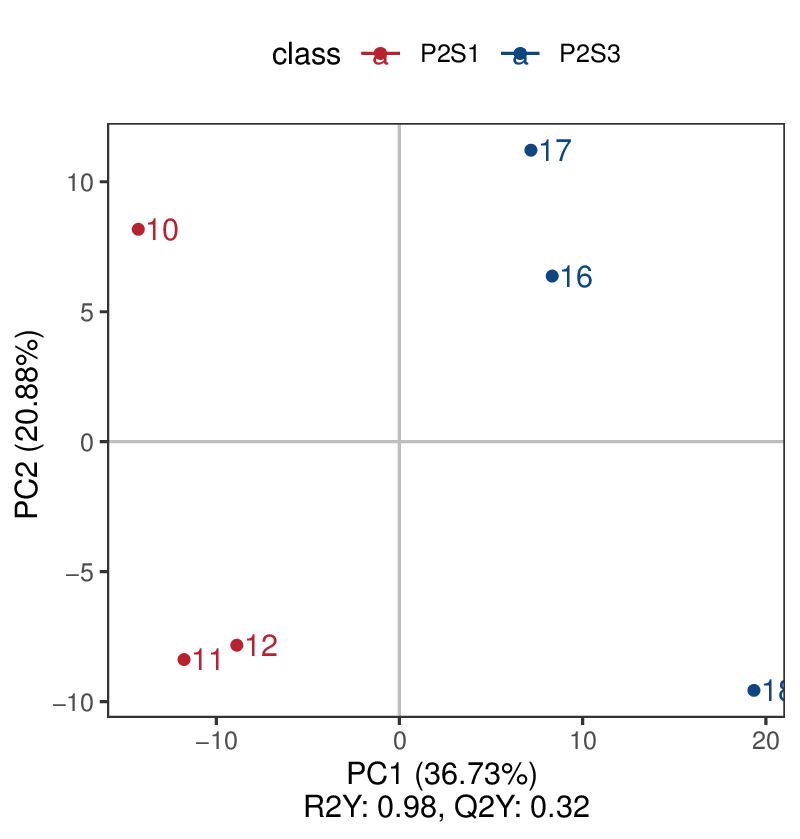


Fig. S3. PLS-DA score diagram of foxtail millet under different Se treatments.


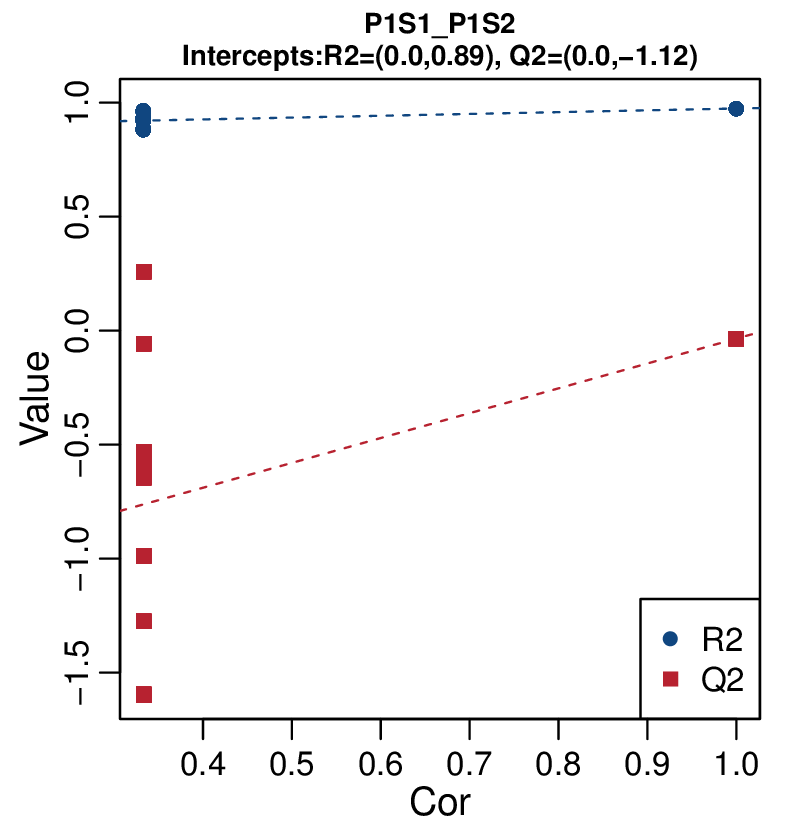

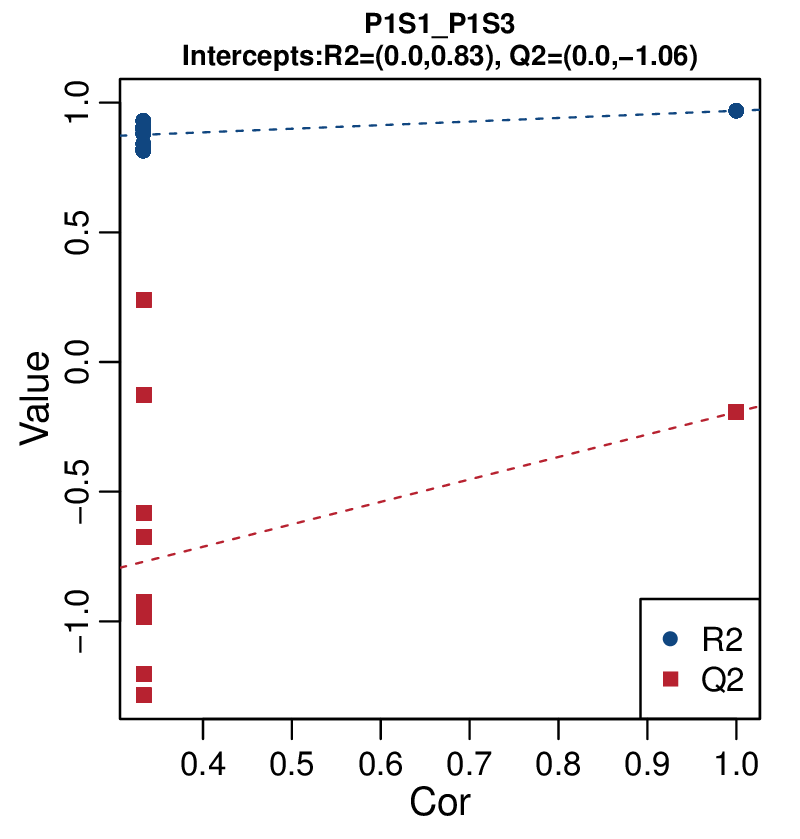

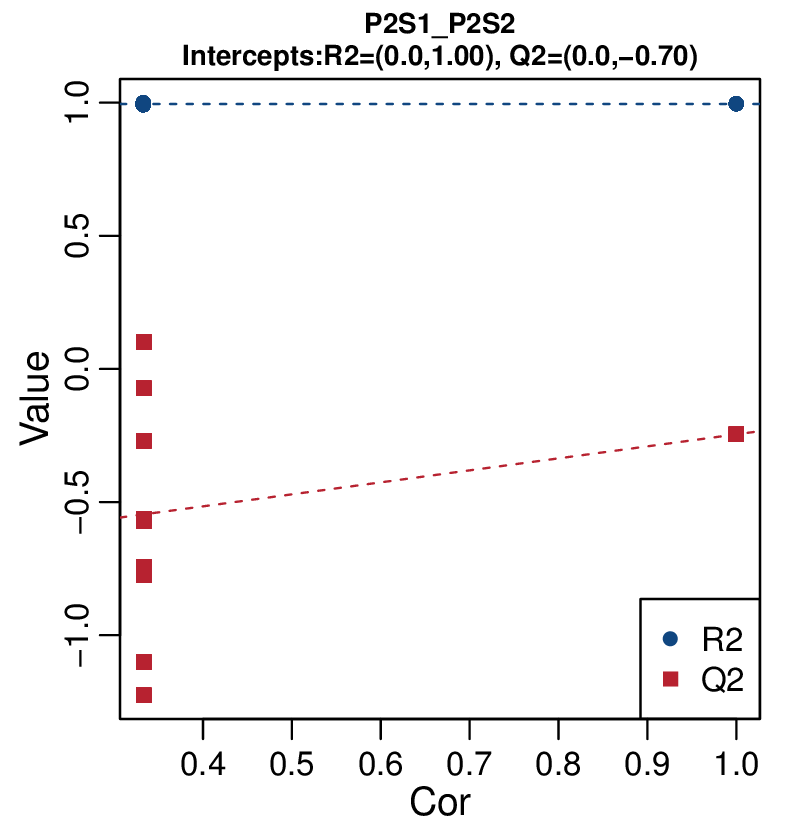

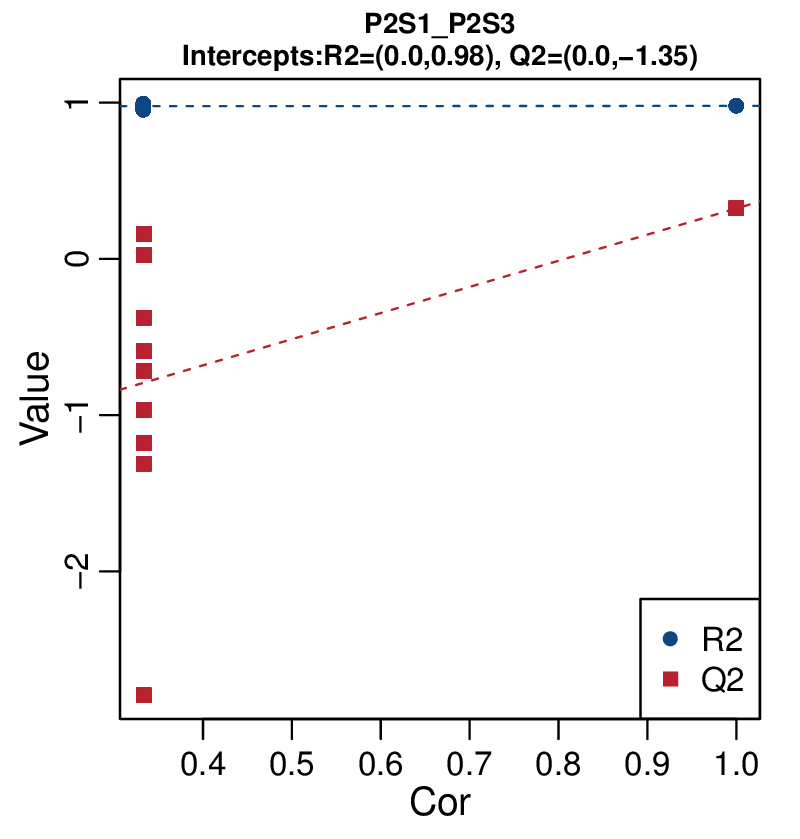


Fig. S4. PLS-DA ordination verification of foxtail millet under different Se treatments.
